# The incidence of radiolucent lines in cemented attune total knee arthroplasty– a retrospective clinical and radiological study

**DOI:** 10.1007/s00402-025-05824-w

**Published:** 2025-03-20

**Authors:** Reza Sorbi, Tilman Walker, Timo A. Nees, Tobias Renkawitz, Babak Moradi, Tobias Reiner

**Affiliations:** 1 Department of Orthopaedics and Trauma Surgery, University Hospital of Schleswig-Holstein, Campus Kiel, Heidelberg, Germany; 2 Department of Orthopaedics, Heidelberg University Hospital, Heidelberg, Germany

**Keywords:** Attune, Total knee replacement, Tka, Radiolucent lines, Aseptic loosening, Patient reported outcome measures, PROM

## Abstract

**Introduction:**

The Attune total knee arthroplasty (TKA) system was introduced with the goal to improve clinical outcome, patient satisfaction and implant survival. The aim of the present study was to investigate the clinical and radiological outcome of the cemented Attune knee and to assess the rate of radiolucent lines (RLL) at a median follow-up of 4 years.

**Materials and methods:**

In this single-center cohort study, we retrospectively evaluated the clinical and radiological results of 165 consecutive patients following cemented TKA with the original design of the Attune knee at a minimum follow-up of two years. Postoperative radiographs were assessed for RLL and clinical outcome was measured using patient reported outcome scores.

**Results:**

A total of 115 patients (127 knees) were available for assessment at a mean follow-up of 47.8 ± 12.9 months. The overall incidence of femoral and tibial RLL was 24% and 26%, respectively. Of the original cohort, two patients had to be revised during the course of the study, resulting in a survival rate of 98.9% at two years with the endpoint “revision for any reason”. Clinical outcome scores improved significantly up to the latest follow-up.

**Conclusions:**

The findings of this study demonstrated good clinical results with significant improvement in pain and knee function and acceptable revision rates at a median follow-up of 4 years. However, a high incidence of periprosthetic RLL was seen in this cohort. No implant showed signs of component loosening, but patients with RLL should be monitored closely and future studies with longer follow-up durations are necessary to investigate the influence of these radiolucencies on the long-term performance of this knee system.

## Introduction

Total knee arthroplasty (TKA) has become a successful and safe treatment option for end stage osteoarthritis of the knee. However, aseptic loosening remains the most common reason for revision surgery in the long term [1–3]. The Attune™ knee arthroplasty system was introduced based on the clinically approved implant design of the P.F.C.™ Sigma^®^ knee in order to improve clinical outcome, patient satisfaction and implant survival. There are different configurations available for the Attune total knee system with a mobile bearing cruciate retaining (CR) and posterior stabilized (PS) rotating platform, as well as a CR, PS and a medial stabilized (MS) fixed bearing tibial platform. In addition, the Attune knee is available as cemented and cementless TKA. Despite good initial results reported in the literature for the cemented Attune TKA [4, 5], some authors have raised concerns regarding higher revision rates due to early debonding and incomplete seating of the Attune tibial component [6, 7]. In a cohort study conducted by Lachiewicz et al., a revision rate of 11.5% for the Attune TKA at an average follow-up period of 30.3 months was reported. Among these revisions, tibial component loosening was observed in 17 out of 19 knees, accounting for 90% of the revised cases [8]. Hoskins et al. reported radiological findings for the cemented Attune TKA with a 23.8% incidence of radiolucent lines at an average follow-up period of 21 months (range 3–51 months) [9].

Various factors can contribute to aseptic implant loosening. While late aseptic loosening is often associated with wear-related problems such as osteolysis due to excessive polyethylene wear, early loosening is usually a consequence of an inadequate initial implant fixation [10]. For example, a poor cementation technique with insufficient cement-interdigitation, leg axis malalignment, or patient specific factors such as high body weight or pronounced osteoporosis, as well as implant specific design characteristics can contribute to an early implant debonding [6]. The detection of radiolucent lines on plain radiographs can be an indication for implant loosening at an early stage, especially if they progress over time [11, 12]. Staats et al. found a significantly higher incidence of radiolucent lines for the Attune knee compared to its predecessor, the P.F.C. knee, 12 months after implantation [13]. In addition, Jaeger et al. observed an increased risk for incomplete seating of the Attune tibial component in an experimental biomechanical study [6]. However, the impact of these findings on the clinical performance of this implant design remain unclear. Therefore, the aim of the present study was to report the clinical, functional and radiological results of the cemented Attune total knee arthroplasty from a non-designer center and to investigate the rate of radiolucent lines at a 4-year median follow-up. Our hypothesis was that cemented TKA using the Attune knee system demonstrated good clinical and functional results with low revision rates and acceptable rates of RLL at short- to mid-term follow-up.

## Materials and methods

This study was a single-center, retrospective cohort study investigating a consecutive cohort of 165 patients, who underwent cemented TKA using the Attune knee system at our institution between February 2014 and December 2017. The local ethics committee approved the study (No. S-804/2019) and written informed consent forms were obtained from all patients. The inclusion criteria were adult patients (> 18 years of age) with severe osteoarthritis of the knee who had received a cemented Attune total knee prosthesis at our department at least 24 months ago. The exclusion criteria were patients who did not consent to participate in the study, legally cared for patients, and patients with comorbidities that impaired the ability to give consent (e.g. dementia, intellectual disability, psychiatric illness) or language barrier. Based on the retrospectively analyzed data set, the patients were either contacted by phone or invited by letter to participate in the study as part of the routine follow-up examination. At the latest follow-up, clinical and radiological parameters as well as postoperative complications were analyzed and validated clinical outcome scores (PROMs) were assessed as described below.

### Operative technique

All surgeries were performed with the patient under spinal or general anesthesia using a medial parapatellar approach and a standard surgical technique according to the manufacturer’s recommendations using the INTUITION™ instrumentation. A femur-first measured resection technique was used in all patients. Distal femoral resection was performed using the intramedullary jig for varus/valgus adjustment and the extramedullary resection guide was used for tibial resection. Extension and flexion gap assessments were performed using the spacer blocks of the Intuition™ instrumentation. According to the intraoperative findings either a cruciate retaining or posterior stabilized femoral component was used. The original design of the cemented Attune fixed bearing tibial component was used in all cases. A tourniquet was applied during cementation and high-pressure pulsatile saline lavage irrigation of the bone was performed prior to cementation. The vacuum mixed bone cement was applied on both the tibial and femoral component as well as on the bone surface via cement gun pressurization. The tibial component and the femoral components were inserted in a single step. The patella was selectively resurfaced, according to the intraoperative findings, if required. The same postoperative rehabilitation protocol with early mobilization and immediate full weight-bearing as tolerated was applied for all patients.

### Clinical and radiographic evaluation

Clinical outcome parameters were determined both before and after surgery using clinical assessments and questionnaires. The following scores were assessed during the latest follow-up: Oxford Knee Score (OKS) [14], Veterans RAND 12 Item Health Survey (VR-12) [15], Knee injury and Osteoarthritis Outcome Score (KOOS) [16], and the clinical and functional American Knee Society Score (AKS) [17]. The AKS score was categorized into four groups: very good result (90 to 100 points), good result (80–89 points), satisfactory result (70–79 points), and unsatisfactory result (< 70 points). Overall patient satisfaction regarding the result of the knee surgery was assessed using a 4-point verbal rating scale (very satisfied, satisfied, neutral, or dissatisfied). Furthermore, subjective patient satisfaction and pain assessment were evaluated using the “Forgotten Joint Score (FJS)” [18], University of California at Los Angeles (UCLA) activity score [19] and the 11-point Numerical Rating Scale (NRS) [20]. All these instruments are validated, reliable and widely used in clinical outcome studies.

The range of motion as well as coronal leg axis alignment were clinically measured using a goniometer. An anatomical valgus angle of 5° to 10° was considered a neutral leg axis, 0° to 4° as mild varus, and < 0° as severe varus deformity. An anatomical valgus angle of 11° to 15° was categorized as mild valgus, and > 15° as severe valgus deformity.

**Fig. 1:**
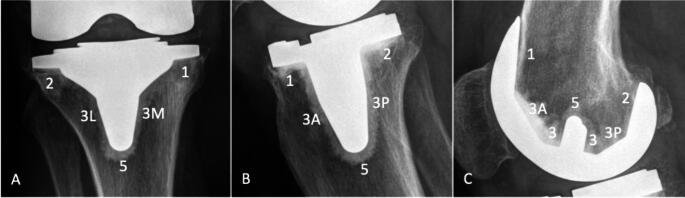
Radiographic assessment of radiolucent lines based on the “Modern Knee Society Radiographic Evaluation System and Methodology for Total Knee Arthroplasty [23]”: (**A**) anteroposterior radiograph of the tibial component (3M: medial side of the keel; 3L: lateral side of the keel); (**B**) lateral radiograph of the tibial component (3A: anterior side of the keel; 3P: posterior side of the keel); (**C**) lateral radiograph of the femoral component (3A: anterior chamfer cut, 3P: posterior chamfer cut)

At the latest follow-up, standard anteroposterior and lateral knee radiographs were taken under fluoroscopy to ensure an appropriate evaluation of radiolucent lines around the tibial and femoral component. Fluoroscopically guided radiography has proven to be superior for the detection of radiolucent lines following unicondylar and total knee replacement when compared to conventional radiographs [21, 22]. Radiolucent lines were defined as periprosthetic radiolucencies less than 2 mm in width with a sclerotic border, that were located at either the cement-bone or cement-implant interface and showed no signs of progression on serial radiographs. Two independent investigators (RS, TR) assessed all radiographs on the basis of the Modern Knee Society Radiographic Evaluation System [23]. Figure 1 illustrates the radiological classification system used for the standardized assessment of radiolucent lines.

### Data management and statistical analysis

All preoperative and postoperative data were documented in an Excel spreadsheet (Microsoft Excel 2019). The graphical representation was done with Excel (Microsoft Excel 2019). Statistical analysis was performed using the program SPSS Statistics for Windows (version 25.0; SPSS IBM Corp., Chicago, IL, USA). Data were evaluated descriptively as arithmetic mean, standard deviation, median, minimum, and maximum. All data were checked for normal distribution using the normality test by D’Agostino and Pearson [24]. The student’s t-test was used to compare means between two groups. Wilcoxon test and McNemar’s test were applied to compare groups when data were not normally distributed. Kaplan-Meier survivorship analysis was performed with revision for any reason as the endpoint. The level of significance was set at *p* < 0.05.

## Results

### Demographic data of the study cohort

In this study, a consecutive series of 165 patients (177 knees) were retrospectively evaluated following TKA with the original Attune knee system. The mean age of the patients at time of surgery was 64.7 ± 10 years. In 51% of the patients TKA was performed on the right side and in 49% of patients on the left side. Seven patients (4%) died regardless of the knee operation during the follow-up period. Eighteen patients (11%) refused to participate in the study and 25 patients (15%) were lost to follow-up. Twelve patients received an Attune TKA on both sides. At the final follow-up, a total of 115 patients (69% female and 31% male) with 127 TKAs were available for clinical and radiological assessment at a mean follow-up of 47.8 ± 12.9 months.

### Survival analysis

The revision-free survivorship of the Attune knee with the endpoint revision for any reason was 98.9% (95% confidence interval [CI]: 96–99%) in this cohort at a mean follow-up of 4 years. No patient had to be revised due to aseptic implant loosening. In one patient, a DAIR procedure with an inlay exchange due to a suspected infection was performed after 14 months. Another patient was revised for patellar resurfacing due to symptomatic retropatellar osteoarthritis after 13 months.

### Clinical evaluation

At the latest follow-up, 84.3% of patients reported to be very satisfied or satisfied with the results of the knee replacement, while 15.7% of patients claimed to be dissatisfied or neutral. 101 patients (87.8%) reported absence of pain at rest and 83 patients (72.2%) reported absence of pain during movement at the latest follow-up. A total of 98 knee joints were clinically examined for range of movement and clinical leg axis assessment using a goniometer, at the last follow-up. The mean postoperative knee flexion angle in the study cohort was 117 ± 17.1 degrees (range 80°– 145°). Postoperative clinical leg axes were within the neutral range in 94.9% of patients, compared to 29.1% of patients preoperatively (p-value < 0.01). The results of the objectively clinical assessment and range of movement are presented in Table 1.

**Table 1:** Results of objective clinical and functional assessment for range of movement and clinical leg axis. SD: standard deviation

	Preoperative (*n* = 127) (Mean ± SD, Range)	Postoperative (*n* = 115) (Mean ± SD, Range)	*p*-value
Range of Movement			
Flexion (Degrees)	108.4 ± 16.4 (35–140)	117 ± 17.1 (80–145)	0.097 (t-test)
Extension deficit (number, percent):			
Full extension	100 (78.7%)	89 (90.8%)	*< 0.0*1
< 10°	16 (12.6%)	5 (5.1%)	(Wilcoxon test)
10° − 15°	6 (5%)	4 (4.1%)	
16° − 20°	5 (3.7%)	0 (0%)	
> 20°	0 (0%)	0 (0%)	
Clinical leg axis (number, percent):			
Neutral	37 (29.1%)	93 (94.9%)	*< 0.0*1
Mild Varus	40 (31.5%)	2 (2%)	(Wilcoxon test)
Mild Valgus	26 (20.5%)	3 (3.1%)	
Severe Varus (< 0°)	13 (10.2%)	0 (0%)	
Severe Valgus (> 15°)	11 (8.7%)	0 (0%)	

Clinical outcome scores demonstrated a significant improvement in AKSS, OKS, UCLA and NRS up to the latest follow-up (p-values < 0.01). The results of patient reported outcome measures investigated in this study are summarized in Table 2.

**Table 2:** Results of clinical outcome scores and numerical rating scale for pain. SD: standard deviation. AKSS: American knee society score. KOOS: knee injury and osteoarthritis outcome score. VR-12: veterans RAND 12 item health survey. UCLA: university of California at Los Angeles

	Preoperative (*n* = 127) (Mean ± SD, Range)	Postoperative (*n* = 115) (Mean ± SD, Range)	*p*-value (t-test)
Oxford Knee Score (OKS)	35.5 ± 8.1 (17–49)	20.7 ± 10.9 (12–47)	*< 0.01*
American Knee Society Score:			
AKSS-O (objective)	42.6 ± 15.5 (3–78)	86.7 ± 19.6 (37–100)	*< 0.01*
AKSS-F (functional)	49.8 ± 22.9 (0–90)	77 ± 15.8 (20–100)	*< 0.01*
Forgotten Joint Score (FJS)		62.7 ± 32.5 (0–100)	
KOOS		72 ± 21.1 (14–100)	
VR-12:			
PCS (physical health component)		38.3 ± 5.4 (29.1–55.1)	
MCS (mental health component)		36.7 ± 5.9 (24.1–52)	
UCLA	4.1 ± 2.4 (1–10)	6.1 ± 2 (1–10)	*< 0.01*
Numerical rating scale (NRS)	7.7 ± 1.2 (3–10)	1.7 ± 0.8 (0–9)	*< 0.01*

### Radiographic results

**Fig. 2:**
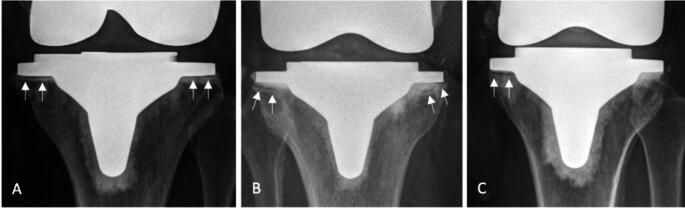
Anteroposterior (AP) radiographs of a 63 years old female patient (**A**), a 71 years old female patient (**B**), and a 54 years old female patient (**C**) of the study cohort following TKA with a cemented fixed bearing cruciate retaining Attune TKA demonstrating the characteristic appearance of tibial radiolucent lines seen in this cohort

**Fig. 3:**
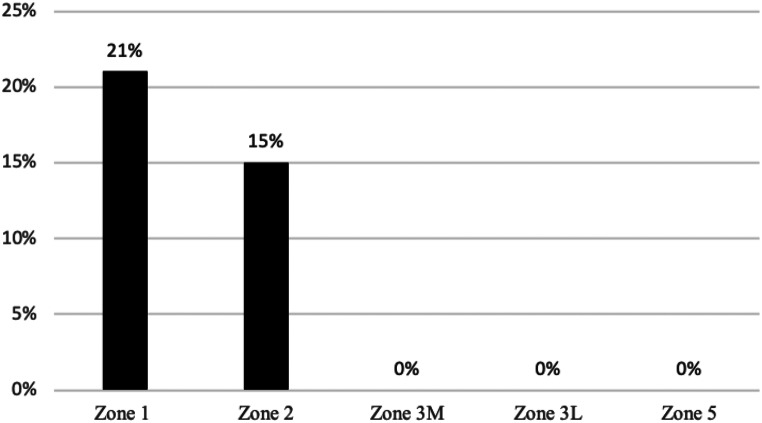
The incidence of tibial radiolucent lines on the anterior–posterior (AP) radiograph assessed according to the “Modern Knee Society Radiographic Evaluation System and Methodology for Total Knee Arthroplasty” [23]

**Fig. 4:**
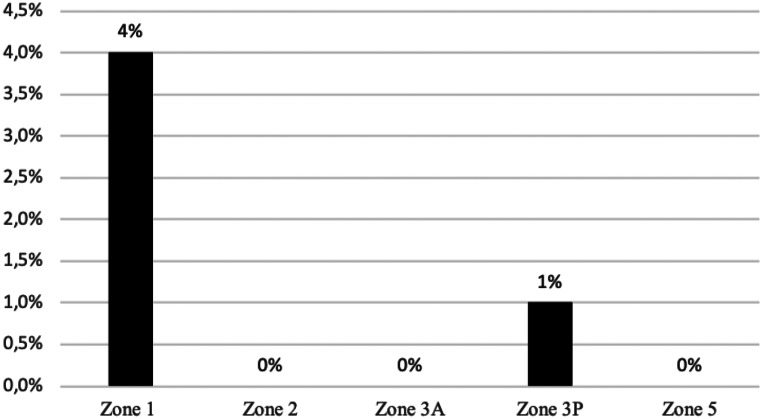
The incidence of tibial radiolucent lines on the lateral radiograph assessed according to the “Modern Knee Society Radiographic Evaluation System and Methodology for Total Knee Arthroplasty” [23]

**Fig. 5:**
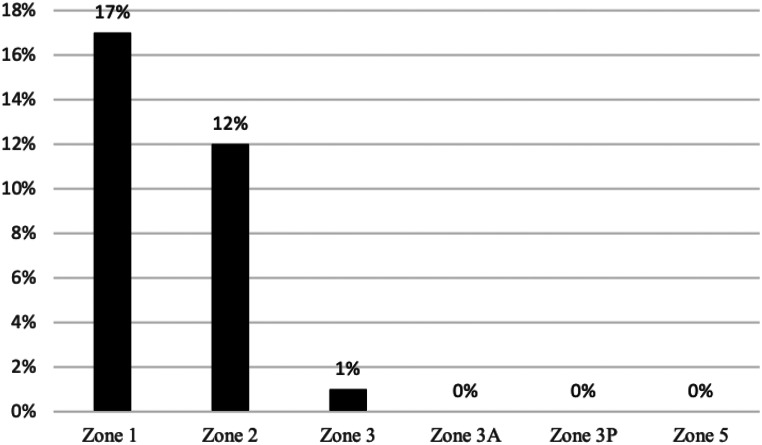
The incidence of femoral radiolucent lines on the lateral radiograph assessed according to the “Modern Knee Society Radiographic Evaluation System and Methodology for Total Knee Arthroplasty” [23]

At latest follow-up, no femoral or tibial component showed radiographic signs of loosening. Radiographs demonstrated an incidence of femoral radiolucent lines of 24% and an incidence of tibial RLL of 26%. RLL were predominantly located in zone 1 and zone 2 of the anterior-posterior (AP) tibial region, comprising 21% and 15% of occurrences. Figure 2 demonstrates the characteristic appearance of tibial RLL seen in this cohort. Lateral radiographs demonstrated RLL predominantly located around the femoral components, with an incidence of 17% and 12% in zone 1 and zone 2, respectively. The distribution of RLL in relation to their location is presented in Figs. 3, 4 and 5.

## Discussion

The goal of introducing a new primary knee system is to improve clinical and functional outcome and to increase patient satisfaction after TKA. Despite good initial results reported in the literature [4, 25, 26], there has been an increasing evidence in recently published studies that the original design of the cemented Attune knee is associated with an increased risk for periprosthetic radiolucencies [27], that could ultimately result in an increased risk of early debonding and implant failure. The etiology of premature tibial debonding remains poorly understood. Proposed hypotheses encompass factors such as cement viscosity, surface finish, diminished stem length, reduced rotational stabilizers, and decreased cement pockets [28]. The aim of this study was to investigate the clinical and functional results of the first Attune TKA performed at our institution and to assess the incidence of radiolucent lines at a 4-year median follow-up.

The rate of RLL in this cohort was relatively high with an overall incidence of 24% for femoral RLL and 26% for tibial RLL. The regions predisposing to a high rate of RLL were identified at the medial and lateral aspect of the tibial baseplate on anterior-posterior radiographs (zone 1 and 2), and on lateral radiographs behind the anterior and posterior flange of the femoral component. The occurrence of radiolucencies at these locations around the tibial and femoral components suggest a potential association with inadequate bone cuts, incomplete component seating during cementation or insufficient cementation technique. Staats et al. [13] hypothesized that the increased number of radiolucent lines in Attune patients is primarily due to technique-related issues, allowing excessive movement during the cement interlocking phase. Additionally, the prosthesis design, particularly the cement pockets, may also contribute to this phenomenon [29]. As a result of reports of early aseptic loosening and tibial debonding, the original design of the Attune tibial component was changed in 2017. The new design of the tibial baseplate (Attune S+) features an undercut cement pocket area and a greater surface roughness of 3.0-6.5 Ra, in order to enhance the mechanical interlock at the cement-implant interface and to increase cement bonding [7]. Van Duren et al. compared the radiological and clinical results of different TKA designs, including the Attune knee, and found no significant differences regarding the incidence of RLL as well as the revision risk between the standard Attune and the Attune S + group [30]. Despite the high incidence of RLL being evident in approximately one quarter of our patients, there was no implant revision due to aseptic loosening in this cohort nor any radiological evidence of implant loosening at final follow-up. Similar results were found by Giaretta et al. [4], who reported radiolucent lines in 22.4% of the investigated Attune knees after a mean follow-up of three years. Two patients had to be revised after 7 and 13 months due to aseptic loosening of the tibial component [4]. Staats et al. [13], reported comparable results in a one-year follow-up study with RLL being present in 35.1% of the knees, however no patient had to be revised as a consequence of aseptic loosening in their cohort. Staats and Giaretta [4, 13] suggested that patients with radiolucent lines should be monitored closely at regular intervals, in spite of good clinical results or absence of symptoms. Several recent studies analyzing the short-term outcome of Attune TKAs reported low revision rates [5, 13, 31, 32]. Prodromidis et al. [27] investigated the incidence of RLL in a recently published meta-analysis, which included a total of 3,861 Attune total knee arthroplasties. They found an overall RLL rate of 21.4%. Notably, the incidence of implant loosening and the revision rate due to aseptic loosening were 1.2% and 0.9%, respectively. These findings are in accordance to the results of our study. O’Donovan et al. [33] also reported a higher incidence of radiolucent lines observed predominantly at the tibial baseplate at the implant–cement interface during a five-year follow-up. Despite this, the revision rate was only 2.2%. This outcome is comparable to data from the Australian Orthopaedic Association National Joint Registry (AOANJR) [34], which reported a 5-year cumulative revision rate of approximately 3% for the Attune knee. Despite the low revision rates, the clinical significance and long-term implications of the relatively high rate of radiolucencies found in our study need to be further investigated. It should be noted that the presence of RLL alone does not necessarily indicate failure or imply an indication for implant revision However, affected individuals should be monitored closely in order to detect potential implant failure in symptomatic as well as asymptomatic patients at an early stage.

Patient satisfaction after TKA is also affected by various additional factors such as excessive patient expectations, persisting pain, the occurrence of postoperative complications, or misalignment of the prosthesis [35, 36]. Bourne et al. [37] presented patient satisfaction rates of 75–89% following total knee replacement. This is in agreement with the results of our study, in which a substantial majority of 84.3% of patients expressed satisfaction with their knee replacement. A significant portion reported relief from rest pain after two years, and many experienced improved walking comfort. Usage of pain relief medication notably declined post-surgery and the majority of participants achieved painless ambulation for extended distances.

The mean range of motion increased from 108.4 degrees of flexion preoperatively to 117 degrees at the last follow-up, marking a substantial improvement, however the difference was not statistically significant. Concurrently, the clinical leg axis improved postoperatively, posited to be linked with reduced pain during passive movement and a normalized leg axis, which promotes knee joint mobility. The findings of Ranawat et al. and White et al. [5, 38] corroborate this association between postoperative pain reduction and improved clinical leg axis alignment, as well as improved knee joint mobility following TKA. The clinical scores and their corresponding outcomes observed in our study collectively displayed favorable results during the a 4-year median follow-up assessment. In a two-year study by Moorthy et al. [25] involving 100 Attune knee replacements, Oxford Knee Scores improved significantly, which is consistent with our findings. The mean UCLA Activity Score in our study significantly increased from 4.1 ± 2.4 points to 6.1 ± 2 points, which is consistent with the findings of Turgeon et al. [26] and Kim et al. [39], emphasizing improved postoperative activity levels following TKA.

There are limitations to our study. On the one hand, the study is limited by its retrospective character and its relatively short follow-up duration. A prospective study design with repetitive radiological examinations and a longer follow-up duration would be helpful to assess the occurrence and progression of periprosthetic RLL over time and to investigate the clinical impact of these radiolucencies on the long-term performance of the implant design. All patients in this study population were followed-up for a minimum of two years after surgery, which ensures an adequate assessment of clinical and functional outcomes, as well as failure rates at a 4-year median follow-up. Another limitation that has to be acknowledged is the lack of a control group that would have enabled a comparison of our radiological findings with those of a clinically proven implant design, such as the PFC Sigma knee. Lastly, a total of 15% of patients were lost and 11% denied the participation in the study. The latter were predominantly elderly patients who refused to travel to our institution for a follow-up examination because of health-related problems and restricted mobility. However, this high drop-out rate represents a limitation and could have potentially biased the results of our study. Despite these limitations, the results of our study support the findings of other authors who reported a high incidence of radiolucent lines associated with the original design of the Attune knee at short- to mid-term follow-up.

## Conclusions

The clinical and radiological findings of this study suggest good clinical and functional results of the cemented Attune knee system with significant improvement in patient reported outcome scores, low rates of implant loosening and acceptable revision rates at a median follow-up of 4 years. However, a relevant proportion of patients demonstrated RLL around the femoral or tibial components, the clinical significance of which remains unclear. Affected individuals should be monitored closely at annual intervals in order to detect debonding or implant failure at an early stage. Further studies with longer follow-up durations are necessary to investigate the natural course of these radiolucencies and their impact on the long-term performance of this knee system.
