# Correction: The incidence of radiolucent lines in cemented attune total knee arthroplasty– a retrospective clinical and radiological study

**DOI:** 10.1007/s00402-025-05902-z

**Published:** 2025-06-26

**Authors:** Reza Sorbi, Tilman Walker, Timo A. Nees, Tobias Renkawitz, Babak Moradi, Tobias Reiner

**Affiliations:** 1 Department of Orthopaedics and Trauma Surgery, University Hospital of Schleswig-Holstein, Campus Kiel, Kiel, Germany; 2 Department of Orthopaedics, Heidelberg University Hospital, Heidelberg, Germany

**Correction: Archives of Orthopaedic and Trauma Surgery (2025) 145:201** 10.1007/s00402-025-05824-w

In the original version of this article, affiliation details for author Reza Sorbi and Babak Moradi were incorrectly given as

^1^Department of Orthopaedics and Trauma Surgery, University Hospital of Schleswig-Holstein, Campus Kiel, Heidelberg, Germany

but should have been

^1^Department of Orthopaedics and Trauma Surgery, University Hospital of Schleswig-Holstein, Campus Kiel, Kiel, Germany

